# Habitual Routines and Automatic Tendencies Differential Roles in Alcohol Misuse Among Undergraduates

**DOI:** 10.3389/fpsyg.2020.607866

**Published:** 2020-12-21

**Authors:** Florent Wyckmans, Armand Chatard, Mélanie Saeremans, Charles Kornreich, Nemat Jaafari, Carole Fantini-Hauwel, Xavier Noël

**Affiliations:** ^1^Laboratoire de Psychologie Médicale, Faculté de Médecine, Université Libre de Bruxelles, Bruxelles, Belgium; ^2^Faculty of Psychology, University of Poitiers, Poitiers, France; ^3^Psychiatric Institute, Universitary Hospital Brugmann, Bruxelles, Belgium; ^4^Faculty of Medicine, University of Poitiers, Poitiers, France; ^5^Research Centre of Clinical Psychology, Psychopathology and Psychosomatic, Université libre de Bruxelles, Brussels, Belgium

**Keywords:** creature of habit scale, questionnaire French translation, alcohol, addiction, compulsivity

## Abstract

There is a debate over whether actions that resist devaluation (i.e., compulsive alcohol consumption) are primarily habit- or goal-directed. The incentive habit account of compulsive actions has received support from behavioral paradigms and brain imaging. In addition, the self-reported Creature of Habit Scale (COHS) has been proposed to capture inter-individual differences in habitual tendencies. It is subdivided into two dimensions: routine and automaticity. We first considered a French version of this questionnaire for validation, based on a sample of 386 undergraduates. The relationship between two dimensions of habit and the risk of substance use disorder and impulsive personality traits was also investigated. COHS has good psychometric properties with both features of habits positively associated with an Obsessive-Compulsive Inventory score. Besides, the propensity to rely more on routines was associated with lower levels of alcohol abuse and nicotine use, suggesting that some degree of routine might act as a protective factor against substance use. In contrast, a high automaticity score was associated with an increased risk of harmful alcohol use. These results demonstrate that the COHS is a valid measure of habitual tendencies and represents a useful tool for capturing inter-individual variations in drug use problems in undergraduates.

## Introduction

A constantly changing environment requires behavioral and decisional adjustments. The repetition of reinforced actions performed within the same context results in behavior triggered automatically in that environment (Dickinson, 1985; Wood and Rünger, 2016). The case of addictive behavior speaks in this regard. Initially motivated by the desire to increase pleasure or to decrease negative emotions (i.e., goal-directed action), influential theories postulate that a large part of addiction-related actions could primarily become habits (i.e., independent of goals to reach; Robbins and Everitt, 1999; Everitt and Robbins, 2016), in the sense that (1) they operate automatically in response to a specific situation, i.e., with little conscious deliberation, and (2) that they are less susceptible to devaluation. However, measuring the relative contribution of habits and goal-directed determinants of a certain action (e.g., having a drink) remains a significant challenge for psychologists (De Houwer et al., 2018) and neuroscientists (Daw et al., 2011; Robbins, 2019).

Several behavioral paradigms, such as the fabulous fruit task (de Wit et al., 2007), the Two-Step Markov Task (Daw et al., 2011), or the appetitive instrumental learning task (Ersche et al., 2016), were used to study the use of habitual and goal-oriented processes in healthy and clinical populations. The results indicate that individuals with compulsive disorders rely more on a habitual mode of response to the detriment of a more flexible one (Gillan et al., 2016; Voon et al., 2017; Wyckmans et al., 2019), notably in alcohol use disorder (AUD; Sjoerds et al., 2013; Sebold et al., 2014, 2017). Additionally, brain imaging studies indicate in compulsive disorder lower engagement of the ventromedial prefrontal cortex and anterior putamen (goal-directed decisions), than the posterior putamen activation (habitual decisions) in alcohol use disorder (Sjoerds et al., 2013), as well as an association between habit formation bias and lower gray matter volumes in caudate and medial orbitofrontal cortices (Voon et al., 2015). Nevertheless, prolonged practice (Dickinson et al., 1995), acute and chronic stress (Otto et al., 2013), as well as exposure to stimulant drugs promote habit formation (Nelson and Killcross, 2006).

A recent self-reported questionnaire, such as the Creature of Habits Scale (COHS; Ersche et al., 2017) could represent an important step to assess inter-individual variations in habitual tendencies. It was designed to differentiate two distinct features of habits: routine behaviors and automatic responses. Routine refers to the execution of familiar action patterns that involve regularity and are likely to be performed daily with a fixed ordered pattern of actions to provide a desired outcome (e.g., efficiency; Clark, 2000; Ersche et al., 2017). Examples of routine are “I generally cook with the same spices/flavorings” or “In a restaurant, I tend to order dishes that I am familiar with.” A central aspect of routines refers to their function purpose (either implicit or explicit), which makes them relatively independent from the immediate environment. Also, routines should continue as long as they give the desired outcome. When the expected does not occur, for instance when a familiar dish does not taste as good as usual, routines are meant to be updated, This functional feature contrasts with automatic responses describing action patterns that are initiated and driven by environmental cues and are not restricted to a fixed temporal pattern, neither involve any kind of deliberation, cognition direction, or dependency on the utility of the outcome (Saling and Phillips, 2007; Ersche et al., 2017). Typical examples of automaticity are “I often find myself finishing off a packet of biscuits just because it is lying there” or “I often find myself running on ‘autopilot,' and then wonder why I ended up in a particular place or doing something that I did not intend to do.” Although considered to be related to habits, routines and automaticity are driven by distinct forces, this is the internal goal in the case of routine and environmental stimuli for automaticity. Interestingly, compulsive behavior was linked with both habit's dimensions, while impulsive tendencies were associated with an increase in automaticity and a decrease in routine (Ersche et al., 2019).

The main advantage of COHS is that it mitigates the weakness of a previous questionnaire, the Self-Report Index of Habits Strength (Verplanken and Orbell, 2003), namely its low focus on context (Sniehotta and Presseau, 2012). Indeed, it is very difficult to ask about stimulus-driven habits without relying on a specific situation. In addition to triggering the behavioral response, the context also triggers the habit's mental representation (Wood and Rünger, 2016), and therefore helps the participant to report them more accurately. Each proposition in the automaticity subscale is therefore contextualized with eating habits (i.e., “when I enter the kitchen”), alimentation being mainly a set of automatic actions (Cohen and Farley, 2008) as well as a universal need.

The aim of the present study was 2-fold: to validate a French version of the COHS, and to investigate the relationship between the propensity to generate routines or automatic responses and the use of alcohol and nicotine in a sample of 386 undergraduates. This research finds its justification in the need to make progress in determining whether habitual automatic responses refer to alcohol misuse, or whether the use is more linked to functional and deliberate routines.

## Materials and Methods

### COHS Translation

The COHS includes 27 forced-choice questions. The participants must choose between 5 propositions, marked from 0 to 4. The score of each scale is obtained by adding the points of its respective questions. The 27 items of the COHS has been translated into French. Based on this translation, two French-English bilingual persons translated it back into English. The discrepancies resulting from this back-translation were discussed and adjustments were made to the French translation of the COHS. The final questionnaire is presented in Supplementary Material.

### Recruitment

We recruited two samples consisting of undergraduate psychology students. The first sample included 100 psychology students from the University of Poitiers (France), while the second sample consisted of 286 students from the Université Libre de Bruxelles (ULB, Belgium). In exchange for their participation, they received course credits. The answers of 21 participants were removed due to their low involvement in the questionnaires. All participants gave informed consent to be part of the experiment. The experiment was approved by the C.H.U. Brugmann Ethics Committee (n° OM 026) and was performed according to the Declaration of Helsinki.

### Questionnaires

After giving their informed consent and some demographic information (age and number of succeeded years from 12 years old), each participant completed the 27 COHS questions on the online LimeSurvey platform. In addition, they complete several validated French version of other questionnaires: harmful alcohol was measured with the Alcohol-Use Disorder Test (AUDIT; French version Cronbach's alpha = 0.87; (Saunders et al., 1993; Gache et al., 2005). Current cigarette smoking was assessed by a yes-no question. The state anxiety was assessed by the Trait-Anxiety Inventory (STAI-YB; French version Cronbach's alpha = 0.89; Bruchon-Schweitzer and Paulhan, 1993). Impulsivity was investigated using the short version of the UPPS Impulsive Behavior Scale (UPPS; French version Cronbach's alpha between 0.7 and 0.84; Whiteside et al., 2005; Billieux et al., 2012). The short version of the Obsessive-Compulsive Inventory (OCIR; French version Cronbach's alpha = 0.86; Zermatten et al., 2006) was used to screen for obsessive behavior and, finally, sensibility to boredom was assessed with the 10 corresponding propositions of the Zuckerman scale (BS Simó et al., 1991; Zuckerman, 1994).

### Statistical Analyses

The original structure of the questionnaire includes 2 factors: the first (“Routine”) contains 16 items, the second (“Automaticity”) 11 items. To assess whether our data fit the same 2-factor solution, a principal component analysis with a varimax rotation was first performed on the first sample. Items with a rotated factor loading lower than 0.35 have been removed. Subsequently, a confirmatory factor analysis was performed on the second sample. All the analyses were made with R studio (v1.1.146), the Lavaan package (Rosseel, 2012), and IBM statistics v26. All data was mean-centered before the analyses.

Following recent recommendations (Awang, 2015), modification indices (MI) were analyzed to assess the redundancy between items. Pairs of items with a MI higher than 10 were evaluated and one was removed if the propositions were considered too redundant. To avoid distorting the original scale, no more than 20% of the original items were removed (Awang, 2015). The validity of each remaining variable was assessed by verifying that each item's factor loading was statistically significant. Regarding the construct validity, since the power of the chi-square increases with sample size, it is unusual for it to be non-significant. It was therefore calculated with several goodness-of-fit indices: The Root Mean Score Error of Approximation (RMSEA), the Standardized Root Mean Square Residual (SRMR), the Comparative Fit Index (CFI), and the Goodness of Fit Index (GFI). An RMSEA and an SRMR between 0.03 and 0.05 is considered a good fit, and between 0.05 and 0.08 an acceptable fit (Awang, 2015). For the GFI and the CFI, a score above 0.9 indicates a good fit. In addition to this confirmatory analysis, Cronbach's alphas were calculated for each subscale and compared to the original scores. A value > 0.7 was considered acceptable. Convergent and discriminant validity was assessed by correlating the two subscales with clinical variables. Finally, *t*-tests (or Mann-Whitney U for non-normal distributions) were used to compare COHS scores between (1) participants with an AUDIT score up to 12 (non-dependent drinkers) vs. those with an AUDIT score of 13 and higher (dependent drinkers; this score being used as a cut-off to discriminate alcohol dependence from excessive consumption; Gache et al., 2005); and (2) nicotine smokers vs. non-smokers.

## Results

### Validation

#### Principal Component Analysis

**Figure 1:**
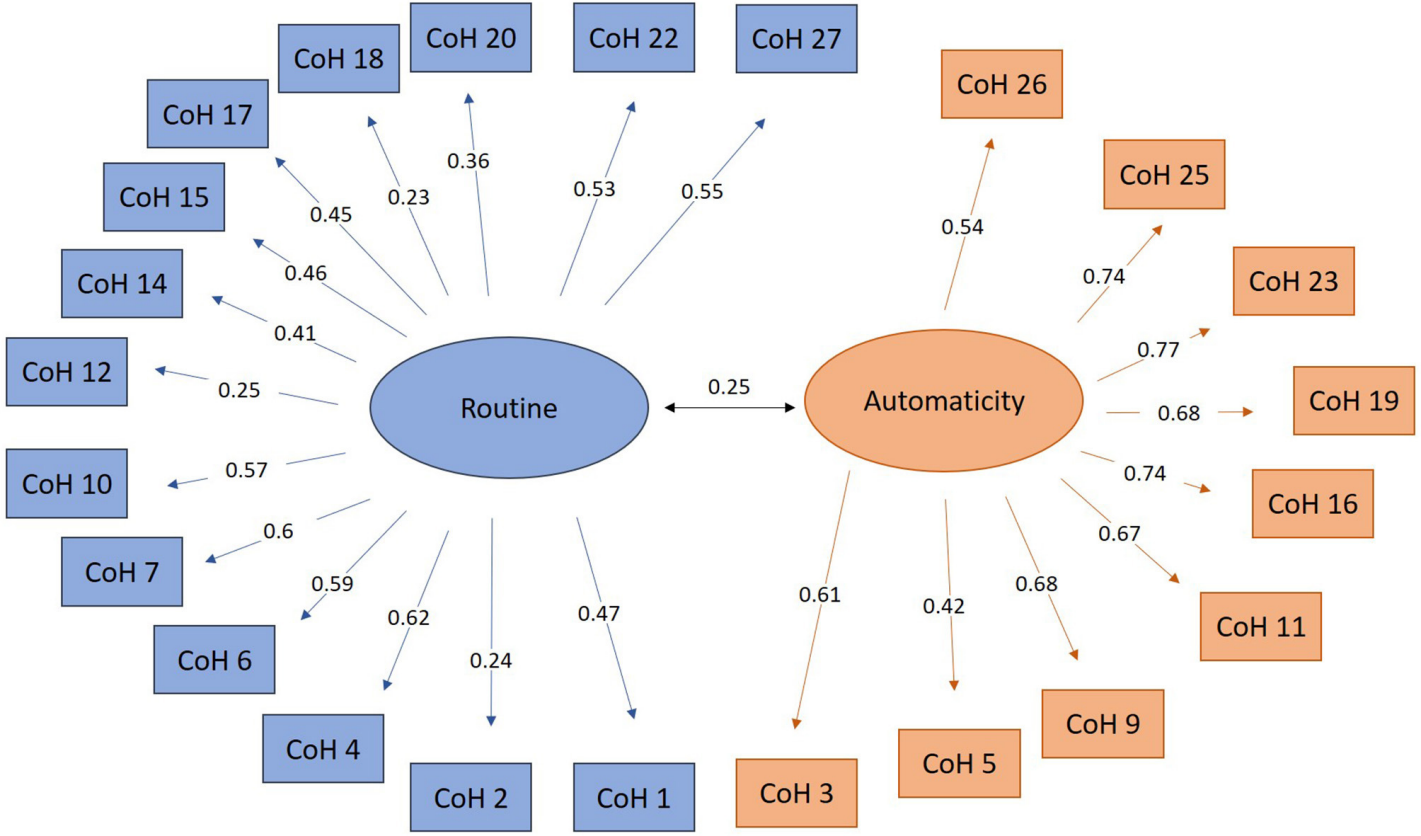
Final rotated factor loading of each item with its respective subscale (first sample) and correlations between routine and automaticity (entire sample).

A principal component analysis was performed on the first sample (*n* = 100). Bartlett's sphericity test, which tests the overall significance of all the correlations within the correlation matrix, was significant for a 2-factor solution [*X*^2^(351) = 836.38, *p* < 0.001] with good sampling adequacy (KMO = 0.66). The rotated component matrix (see Figure 1) showed that the 21st (automaticity) and the 24th (routine) items had a low correlation with both subscales (< 0.2) and were therefore deleted.

#### Confirmatory Factor Analysis

A confirmatory factor analysis covering all items except the 21st and the 24th was performed on the second sample (*n* = 265). The MI showed that four pairs of items were too redundant (items 6 and 13; items 8 and 11; items 19 and 21; items 12 and 17). We analyzed the pair and decided to remove the pair with the lowest factor load (respectively, questions 13; 8; 21; and 17), because they were semantically very similar. The rotated factor loads of the remaining variables with the subscales were significant (*p* < 0.001). The construct validity measures were all on their respective cut-off score (RMSEA = 0.04; SRMR = 0.06; CFI = 0.88; GFI = 0.9) and were close to those obtained by Ersche et al. (2017).

#### Internal Validity

Cronbach's alpha was calculated over the entire sample for the final version of the questionnaire. The “routine” and “automaticity” subscales both showed good Cronbach's alphas (0.73 and 0.8, respectively), close those initially observed by Ersche et al. (2017).

### Clinical Questionnaires

The descriptive scores for each variable are presented in Table 1. The mean severity score for alcohol consumption, measured by the AUDIT, was low and correspond to low-risk use.

**Table 1:** Descriptive statistics of each measure.

**Variable**	***N***	**Mean (SD)**
**General**
Age	365	20.27 (4.83)
Study level	265	12.49 (1.65)
**COHS scores**
Routine	365	49.75 (8.25)
Automaticity	365	26.68 (8.04)
**Clinical profile**
UPPS	265	48.69 (8.15)
State-anxiety	265	49.68 (8.52)
Boredom susceptibility	265	2.92 (1.91)
OCIR	265	23.25 (10.92)
AUDIT	265	5.61 (6.3)

**Figure 2:**
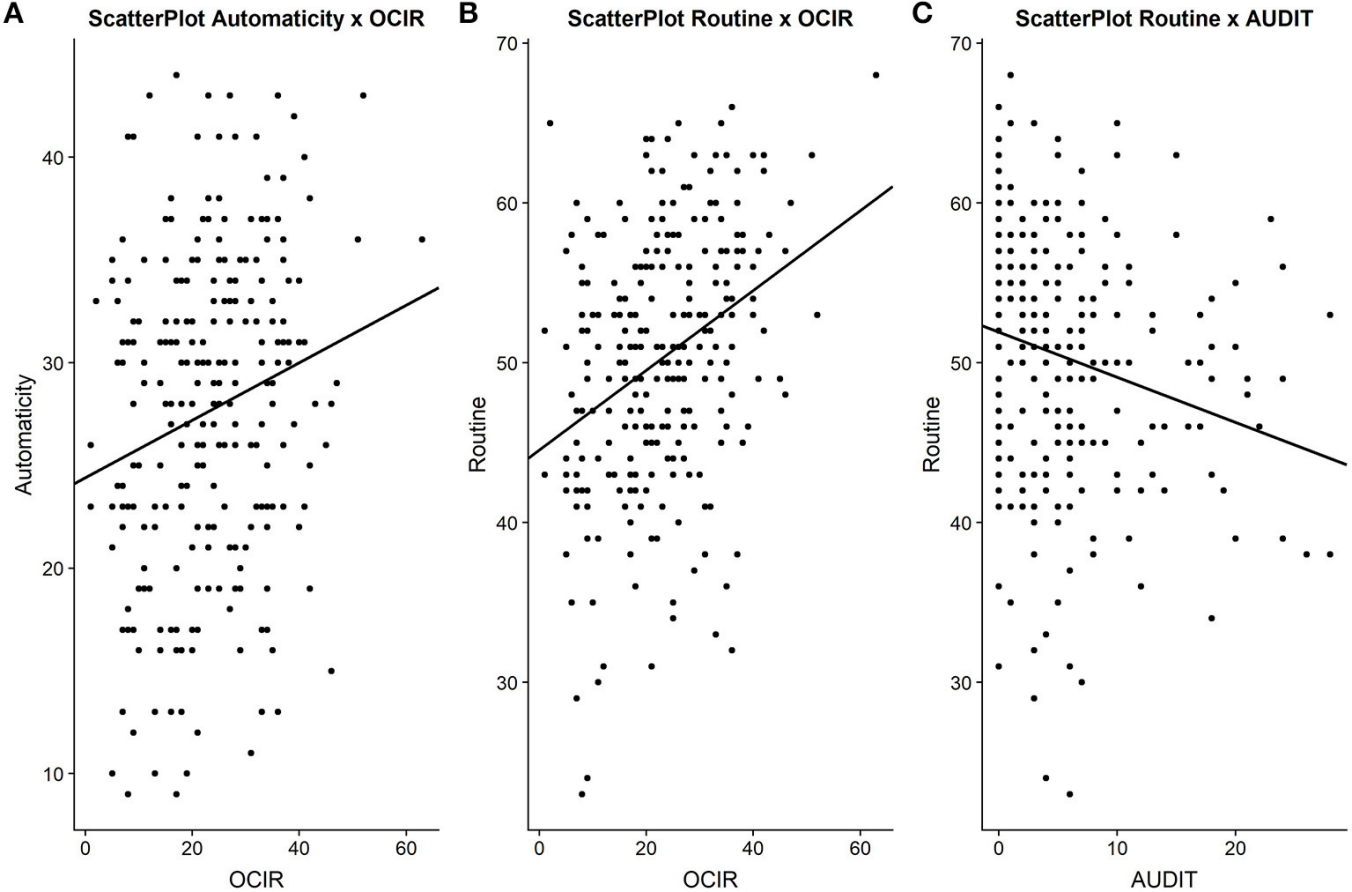
Scatterplots representing correlations between **(A)** Automaticity and Obsessive-Compulsive Inventory (OCIR) scores, **(B)** Routine and OCIR scores, and **(C)** routine and Alcohol-Use Disorder Test (AUDIT) scores.

Correlations were performed between each subscale, AUDIT score and clinical profile (see Table 2 and Figure 2). As was the case in the original COHS, routine and automaticity were weakly but significantly correlated (Pearson's *r* = 0.25, *p* < 0.001).

**Table 2:** Pearson's correlation between the two subscales and the Obsessive-Compulsive Inventory (OCIR), Susceptibility to Boredom (BS), UPPS Impulsive Behavior Scale (UPPS), Trait-Anxiety Inventory (STAI-YB), Alcohol Use Disorder Test (AUDIT) scores, as well as the age and the number of succeeded years from age 12).

		**OCIR**	**BS**	**UPPS**	**STAI-YB**	**AUDIT**	**Study level**	**Age**
Automaticity	Pearson Correlation	**0.197**	−0.053	−0.082	0.09	0.066	–**0.199**	−0.049
	*p*-value	**0.004**	0.394	0.181	0.147	0.285	**0.003**	0.346
	*N*	**265**	265	265	262	265	**265**	365
Routine	Pearson correlation	**0.337**	–**0.319**	–**0.132**	0.073	–**0.22**	−0.113	−0.043
	*p*-value	**<** **0.001**	**<** **0.001**	**0.031**	0.237	**<** **0.001**	0.066	0.411
	*N*	**265**	**265**	**265**	262	**265**	265	365

*The significant correlations are indicated in bold. The Holm-Bonferroni correction for multiple comparisons was performed on significant results to avoid inflating the false positive rate*.

In addition, females reported being significantly less exposed to nicotine (*X*^2^ = 4.9, *p* = 0.03), and alcohol (*X*^2^ = 5.62, *p* = 0.02) than males. The AUDIT score significantly correlated with the routine (Pearson's *r* = −0.22, *p* < 0.001; see Table 2) and the boredom susceptibility (Pearson's *r* = 0.26, *p* < 0.001) scores, but not with the automaticity score (*p* > 0.05). Routine score was significantly lower in dependent alcohol drinkers than in non-dependent drinkers (*U* = 3,066, *p* = 0.01) and lower in nicotine smokers than in non-smokers (*U* = 2,150, *p* < 0.001). The automaticity score was significantly higher in dependent drinkers than in non-dependent drinkers (*U* = 3,027, *p* = 0.01) and did not significantly differ according to nicotine consumption (*p* > 0.05). The mean scores for each group are shown in Table 3.

**Table 3:** Mean scores for both scales separated by sex and substance use.

	**Mean routine score (SD)**		**Mean automaticity score (SD)**	
**AUDIT score (n)**	**Dependent drinkers (36)**	**Non-dependent drinkers (229)**	**Dependent drinkers (36)**	**Non-dependent drinkers (229)**
	**47.69 (6.76)**	**50.76 (8.21)**	**30.67 (6.95)**	**27.19 (7.9)**
Smokers (n)	**Smokers (32)**	**Non-smokers (233)**	Smokers (32)	Non-smokers (233)
	**45.44 (6.48)**	**51.02 (8.06)**	25.63 (8.01)	27.94 (7.7)
Gender (n)	Female (219)	Male (46)	Female (219)	Male (46)
	50.78 (8.11)	48.26 (7.68)	28.05 (7.73)	25.85 (7.75)

*Scores in bold are significantly different*.

## Discussion

The aim of this study was 2-fold: to validate a French version of the Creature of Habit Scale (COHS) and to estimate the association between the tendency to act routinely and/or automatically and substance use (tobacco and alcohol) among undergraduates. First, COHS has good psychometric properties with two distinct features of habits, namely routine behaviors and automatic responses, both of which were associated with an Obsessive-Compulsive Inventory score (OCIR) (Zermatten et al., 2006). Second, the propensity to rely more on routines was associated with lower levels of alcohol abuse and smoking, suggesting that some degree of routine might act as a protective factor against drug use in a sample of undergraduates. In contrast, a high automaticity score was associated with an increased risk of experiencing harmful use of alcohol. These results are now discussed.

Measures of the fit attested that the French translation exhibits a solid construct with good psychometric properties, similar to the original English version (Ersche et al., 2017). Of the 27 original items, four were removed (two with low correlation to other questions and two deemed redundant; see Supplementary Material). According to the criteria described by recent guidelines (Awang, 2015), our translation remains consistent with its original version and exhibited good fit measures, as well as acceptable and good internal consistency, respectively, for sub-scales of routine and automaticity. In addition, the expected associations between the COHS subscales and clinical scores indicate good external consistency. Indeed, as in previous studies (Ersche et al., 2017, 2019), we found an association between a compulsivity score (Zermatten et al., 2006) and the two COHS subscales, thus confirming the past empirical results showing individuals with a compulsive tendency relied strongly on a habitual mode of action (Voon et al., 2015; Gillan et al., 2016, 2019). Additionally, we found that the impulsivity was inversely proportional to routine tendencies, which is consistent with the proposition that goal-striving individuals managing their behaviors with consideration for the consequences of their actions in mind are less inclined to allow environmental stimuli to take over control, a phenomenon related to impulsivity (Lanza and Drabick, 2011; Ersche et al., 2019). Moreover, the non-significant correlations between the two subscales and age, as well as the weak positive correlation between routine and automaticity were similar to the results of the original scale (Ersche et al., 2017, 2019). Finally, the routine score correlates negatively with the boredom susceptibility score of the sensation-seeking scale (Zuckerman, 1994), indicating that the more people are susceptible to boredom, the less they develop functional habits, a phenomenon (boredom susceptibility) known to promote the development of addictive behavior (Orcutt, 1984).

Regarding the expected relationship between substance use and automaticity, we found that dependent drinkers had higher automaticity scores than non-dependent drinkers. These results, along with the positive correlation between automaticity and compulsive tendencies, suggest that high automaticity could represent a risk factor in the development of compulsive disorders, such as addiction. Consistently, the habit sensitization theories (Everitt and Robbins, 2016; Robbins, 2019) postulates that addictions are highly dependent on contextual cues and are triggered almost unconsciously, representing two key characteristics of automatic behavior. In addition, a strong reliance on contextual cues has been reported in alcohol use disorder by empirical paradigms, such as the Markov Two-Step Task or the Pavlovian to Instrumental Transfer Task (Sebold et al., 2014, 2017; Doñamayor et al., 2018).

On the other hand, the propensity to develop routines was negatively correlated with the risk of alcohol misuse and was lower among smokers and dependent alcohol drinkers than non-smokers and non-dependent drinkers, suggesting that routine behaviors can be somewhat protective in undergraduates. These results could be explained by certain sample characteristics. Some studies have suggested that alcohol binge drinking and alcohol use disorder may be related to habits, a phenomenon related to weakened goal-directedness and top-down control (Sebold et al., 2014; Doñamayor et al., 2018). However, the picture is more nuanced when considering undergraduates with excessive drinking. Indeed, a recent study found no association of goal-directed and habitual control with alcohol consumption in young adults (Nebe et al., 2018). Additionally, the use of psychoactive substances in students could be primarily motivated by expected outcomes (i.e., positive expectations), such as avoiding boredom (Biolcati et al., 2018) or seeking sensations (Hamdan-Mansour et al., 2018), two personality traits associated with a lower level of routine (Ersche et al., 2019). It should be noted that we also found some support for a positive relationship between alcohol misuse (AUDIT) and susceptibility to boredom. Taken together, based on the current results, we hypothesize that routine tendencies may protect against stimulus-driven (automaticity) substance use (e.g., tobacco and alcohol) at a young age. In addition, research focusing on people with a longer history of AUD (e.g., patients seeking treatment) is warranted to determine whether automaticity over routines is more involved in this late stage of addiction.

The present study is not without limitations. First, in order to contain the duration of the examination, the degree of nicotine dependence was not recorded, thus preventing the detection of inter-individual differences between smokers. Indeed, in order to clarify the association between daily routines, automatic behaviors and nicotine addiction, additional information regarding the age of smoking's onset, the duration of smoking and the severity of nicotine dependence should be examined in other cross-sectional and prospective studies. Second, our sample included only undergraduates, which limits the generalization of the results. Indeed, although we observed for a minority of participants the presence of harmful behaviors (i.e., AUDIT score > 12; Gache et al., 2005) that could lead to a state of addiction, most of them could be transitory because they are strongly related to the stages of life (Substance Abuse Mental Health Services Administration, 2019). Further research including sub-clinical and clinical populations with a broader age and severity range of substance use is needed to better characterize the relationship between compulsive use of psychoactive substances and habitual behaviors. Finally, the COHS lets us unaware of an important aspect of compulsive actions, namely resistance to devaluated actions (Ersche et al., 2017). Indeed, by definition, habits refer to inflexible and persistent actions despite the degradation of the reinforcer, which has been studied mainly by behavioral paradigms (de Wit et al., 2007; Ersche et al., 2016). A recommended approach to fully apprehend the mechanisms of compulsivity is to combine several methodologies, namely, the COHS and behavioral paradigms.

To conclude, the present French adaptation of the Creature of Habit Scale (Ersche et al., 2017) is a valid measure of inter-individual variations in habitual tendencies and represents a useful tool for capturing important personality traits involved in compulsive disorders.

## Data Availability Statement

The datasets presented in this study can be found in online repositories. The names of the repository/repositories and accession number(s) can be found at: https://osf.io/wbgr7/.

## Ethics Statement

The studies involving human participants were reviewed and approved by C.H.U. Brugmann Ethics Committee (n° OM 026). The patients/participants provided their written informed consent to participate in this study.

## Author Contributions

FW: conceptualization, methodology, formal analysis, investigation, data curation, writing—original draft, and visualization. AC: conceptualization, methodology, investigation, writing—review, and editing. MS, NJ, CF-H, and CK: writing—review and editing. XN: conceptualization, writing—original draft, writing—review and editing, and supervision. All authors contributed to the article and approved the submitted version.

## Conflict of Interest

The authors declare that the research was conducted in the absence of any commercial or financial relationships that could be construed as a potential conflict of interest.
