# Supplementary material for: Habitual Routines and Automatic Tendencies Differential Roles in Alcohol Misuse Among Undergraduates
Source: Front Psychol. 2020 Dec 21;11:607866. doi: 10.3389/fpsyg.2020.607866 (PMC7779402; doi:10.3389/fpsyg.2020.607866)
Supplement: Supplementary file 1 [file Table_1.DOCX]

# Supplementary material

## French questions in the Creature of Habit Scale

The participant must choose one answer among 5 possibilities: “Total désaccord” (0), “Désaccord moyen” (1), “Pas d’avis” (2), “Accord moyen” (3), “Total accord” (4), rated from 0 to 4 points. Points are then summed for each subscale.

| Item number | Subscale | Question |
| --- | --- | --- |
| 1 | Routine | J’aime garer ma voiture ou mon vélo toujours au même endroit. |
| 2 | Routine | Je cuisine généralement avec les mêmes épices et goûts. |
| 3 | Automaticity | Lorsque je passe un plateau de biscuits ou de gâteaux, je ne peux pas résister à en prendre un. |
| 4 | Routine | J’ai tendance à aller au lit plus ou moins à la même heure. |
| 5 | Automaticity | Je prends souvent quelque chose à grignoter lorsque je suis en mouvement (par exemple, lorsque je conduis, quand je marche dans la rue ou lorsque je surfe sur le net). |
| 6 | Routine | Je préfère rester dans ma zone de confort plutôt que prendre des risques. |
| 7 | Routine | Je fais les choses dans le même ordre tous les matins (par exemple, me lever, aller à la salle de bain, prendre un café, …). |
| 8* | Automaticity | Manger des chips ou des biscuits jusqu’à ce que le paquet soit vide est typique de moi. |
| 9 | Automaticity | A chaque fois que j’entre dans la cuisine, j’ouvre le frigo pour voir s’il n’y a pas quelque chose à manger. |
| 10 | Routine | J’essaie toujours de m’assoir à la même place dans un bus, au cinéma ou au restaurant. |
| 11 | Automaticity | Il m’arrive souvent de terminer un paquet de biscuits juste parce qu’il est posé là devant moi. |
| 12 | Routine | J’ai l’habitude d’acheter le même type de nourriture dans le même magasin. |
| 13* | Routine | Je préfère faire des choses que je connais bien plutôt que d’explorer de nouvelles expériences. |
| 14 | Routine | Au petit déjeuner, Je mange généralement la même chose tous les jours. |
| 15 | Routine | J’aime bien mes habitudes. |
| 16 | Automaticity | J’ai pour habitude de terminer une journée de travail par du grignotage. |
| 17* | Routine | Au restaurant, j’ai tendance à commander ce que je connais bien. |
| 18 | Routine | Je suis une personne qui déteste quand on annule à la dernière minute. |
| 19 | Automaticity | Il m’arrive de manger sans y penser, par habitude. |
| 20 | Routine | Je m'assoie souvent à la même place à table |
| 21* | Automaticity | Je fonctionne parfois en pilotage automatique, et ensuite je me demande ce que je suis en train de faire. |
| 22 | Routine | Je respecte toujours un certain ordre quand je prépare le repas. |
| 23 | Automaticity | Regarder la télévision me donne une envie irrésistible de manger |
| 24** | Routine | J’ai tendances à utiliser le plus longtemps possible une version de logiciel que je connais plutôt que de tester une version plus récente. |
| 25 | Automaticity | Il m’arrive souvent d’ouvrir une armoire pour prendre quelque chose à manger. |
| 26 | Automaticity | J’ai tendance à manger quelque chose lorsque je me sens stressé. |
| 27 | Routine | Je me sens bien lorsque les choses se répètent. |
| Supplementary material 1. Creature of Habits Scale French translation. *Questions removed in the French translation due to their redundancy. **Question removed due to their low factor loading. | | |
